# Gross Karyotypic and Phenotypic Alterations among Different Progenies of the *Candida glabrata* CBS138/ATCC2001 Reference Strain

**DOI:** 10.1371/journal.pone.0052218

**Published:** 2012-12-20

**Authors:** Oliver Bader, Alexander Schwarz, Eefje A. Kraneveld, Marut Tangwattanchuleeporn, Pia Schmidt, Mette D. Jacobsen, Uwe Gross, Piet W. J. De Groot, Michael Weig

**Affiliations:** 1 Institute for Medical Microbiology and German National Reference Center for Systemic Mycoses, University Medical Center Göttingen, Göttingen, Germany; 2 Department of Preventive Dentistry, Academic Centre for Dentistry Amsterdam, University of Amsterdam and VU University Amsterdam, Amsterdam, The Netherlands; 3 Aberdeen Fungal Group, Institute of Medical Sciences, Aberdeen, United Kingdom; 4 Regional Center for Biomedical Research, Albacete Science & Technology Park, University of Castilla – La Mancha, Albacete, Spain; New Jersey Medical School, University of Medicine and Dentistry of New Jersey, United States of America

## Abstract

Genomic plasticity is a mechanism for adaptation to environmental cues such as host responses and antifungal drug pressure in many fungi including the human pathogenic yeast *Candida glabrata*. In this study we evaluated the phenotypic and genotypic stability of the world-wide used *C. glabrata* reference strain CBS138/ATCC2001 under laboratory conditions. A set of ten lineages of this wild type strain and genetically modified progenies were obtained from different scientific laboratories, and analyzed for genotypic and phenotypic alterations. Even though the derivates were indistinguishable by multi locus sequence typing, different phenotypic groups that correlated with specific karyotypic changes were observed. In addition, modifications in the adherence capacity to plastic surface emerged that were shown to correlate with quantitative changes in adhesin gene expression rather than subtelomeric gene loss or differences in the number of macrosatellite repeats within adhesin genes. These results confirm the genomic plasticity of *C. glabrata* and show that chromosomal aberrations and functional adaptations may occur not only during infection and under antimicrobial therapy, but also under laboratory conditions without extreme selective pressures. These alterations can significantly affect phenotypic properties such as cell surface attributes including adhesion and the cell wall carbohydrate composition and therefore, if unnoticed, may adulterate the outcome of genetic studies.

## Introduction

After *Candida albicans, Candida glabrata* is the yeast second most frequently isolated from patients suffering from systemic candidiasis [1], [2]. *C. glabrata* shows a high intrinsic resistance to commonly used azole antimycotics [3], and disseminated human infections are associated with a high mortality.

*C. glabrata* has a haploid genome consisting of 13 chromosomes, and although this yeast possesses orthologs of many known mating genes, it primarily uses a clonal mode of reproduction [4], [5]. In the year 2004 the genome sequence of the *C. glabrata* isolate CBS138/ATCC2001 has become available [6] and valuable molecular tools, such as auxotrophic parental strains based on the *C. glabrata* isolate CBS138/ATCC2001 have been developed [7], [8] to study the pathogenicity of this organism. As the cell wall of *C. glabrata* determines adhesion to and subsequent interaction with host tissues, and as it is considered an attractive target for antimycotic therapy, this organelle has been a focus of scientific interest during the last decade [9], [10].

During the course of infection and under antimycotic drug pressure, dynamic genetic alterations have been observed in clinical isolates of *C. glabrata* and *C. albicans*, leading to increased drug resistance during therapy and subtle adaption of adhesion to host tissues. These molecular dynamics include the formation of isochromosomes, the duplication or loss of entire or partial chromosomes, ectopic homologous recombination, multiplication of megasatellite sequences, as well as epigenetic chromatin-based silencing [11], [12], [13]. Large-scale genome comparisons show that both, *C. albicans* and *C. glabrata*, possess a highly flexible genome [14]. Combined with a very dynamic cell wall structure, this confers an adaptive plasticity when invading adverse environments such as the human host [15]. In clinical isolates of *C. glabrata*, karyotypic changes in the two largest chromosomes L and M have been described frequently in the literature [16], [17], [18], [19], [20], [21]. However, recent analyses of clinical *C. glabrata* strains also revealed size heterogeneity of additional chromosomes based on reciprocal chromosomal translocations and recombination within tandem arrays of genes with internal repeats [22], [23]. Interestingly, in *C. glabrata* gene tandems often encode cell wall proteins and many genes that encode cell wall proteins are localized at highly dynamic subtelomeric regions [24], [25]. Consequently, it has been suggested that under high selective pressure chromosomal alterations in subtelomeric regions might affect phenotypic properties [22], especially cell wall-mediated functions.

Since Muller *et al.* noticed that the CBS138-based histidine auxotrophic strain *C. glabrata* dH1 [8], which is commonly used for genetic experiments, harbors a chromosomal rearrangement involving ChrK [22], we raised the question how stable karyotypes and phenotypes are maintained in the parental strain CBS138/ATCC2001 and its progenies under laboratory conditions. The occurrence of aneuploidies and other chromosomal changes during *in vitro* mutagenesis is a recognized technical problem in the diploid fungus *C. albicans*, and influences the outcome of genetic studies [26], [27], [28], [29].

In this study, we have analyzed the reference strain *C. glabrata* CBS138 and nine of its progenies obtained from independent research laboratories working with this strain. In addition, the karyotypes and cell-wall related phenotypes of a collection of eight different *C. glabrata* CBS138-derived mutants, that were generated for research purposes in the past (including strain dH1), were compared. We show that there is significant genomic and phenotypic diversity of this reference strain. This study adds new information about profound difficulties in the inter-laboratory comparability of phenotypes e.g. in generated mutant strains of *C. glabrata*.

## Materials and Methods

### Strains and Media

Isolates used in this study (Table 1) were stored using Cryobank™ (MAST Group, Bootle, UK), propagated on YPD agar plates (1% [w/v] yeast extract, 2% [w/v] peptone, 2% [w/v] dextrose, 1.5% [w/v] agar) and experiments were started from overnight cultures grown in liquid YPD at 30°C or 37°C under shaking, unless stated otherwise.

**Table 1:** Strains used in this study.

Strain	Parental strain	Reference
CBS138/1	–	CBS
CBS138/2	CBS138/1	this study
CBS138/3	CBS138/1	this study
CBS138/4	CBS138/1	this study
CBS138/5	CBS138/1	this study
CBS138/6	CBS138/1	this study
CBS138/7	CBS138/1	this study
CBS138/8	CBS138/1	this study
CBS138/9	CBS138/1	this study
CBS138/10	CBS138/1	this study
2001TU	CBS138/1	[7]
2001HTU	CBS138/1	[7]
dH1	Δht	[8]
dHT6	Δhtu	[8]
Δ*phr1*	dH1	[8]
Δ*kex2*	dH1	[33]
Δ*gas1*	dH1	[8]

### Pulsed-field Gel Electrophoresis (PFGE)

Chromosomal-DNA-containing agarose plugs were prepared as described previously [30]. PFGE was carried out using the CHEF-DR-II-system (Bio-Rad, Munich, Germany) for 22 h at 200 V with pulse-times from initially 60 s to finally 120 s at a fixed angle of 120° in 1% [w/v] agarose (TAE, pH 8.5) at 14°C. Gels were stained in 50 µg•ml^−1^ ethidium bromide for 20 min followed by destaining in deionized water for 20 min.

### PCR, MLST and Quantitative Real-time PCR

Gene selection, PCR amplification and DNA sequencing for Multi Locus Sequence Typing (MLST) were performed as described previously [31].

For PCRs on genomic DNA, whole genomic DNA was isolated by conventional phenol-chloroform extraction of cells harvested from overnight cultures and broken using a Fast Prep 120 machine (BIO101/Savant). PCR products (Table 2) were amplified under primer-specific conditions, varying annealing temperatures from 62–68°C and extension times from 60 to 90 s, using taq polymerase (Roche, Mannheim, Germany).

**Table 2:** Oligonucleotides.

Target name	Direction	Sequence	Product size
*EPA12* (CAGL0M00132g)	Fwd	CGGGGTCGAGACCGACCTCA	418 bp
	Rev	GCTCGGTGATCAAGCCATCGACA	
*EPA21* (CAGL0D06732g)	Fwd	AGAGCCACCCACAAGGGTGT	227 bp
	Rev	CGCTGTCGAGATGTAACGGGCC	
*PWP6* (CAGL0M14069g)	Fwd	TTGTTCCCACCTGGGGGCGA	397 bp
	rev	GCCGTCACCACCACCGTTGT	
CAGL0M00110g	fwd	ACACGAATGGCACGGTGAAAGCA	201 bp
	rev	TGTCAACGGTAACAATCCCAGCGG	
CAGL0D00110g	fwd	GGCTTGCCGTTGGAGTCGGT	482 bp
	rev	ACGAACGCTGACGGCAGTGT	
repeat region of *AWP2*	fwd	GAACTGGGATTTTCGGAGACCG	1119 bp
	rev	GTTCTCAACGTGGGATTCCAAATC	
repeat region of *AWP5*	fwd	CCGAGTCTAATGGATTGGGATCTGA	1662 bp
	rev	CCACTATCTTGGTGTCAAC	

Oligonucleotide sequences for qPCR analysis of adhesin genes *AWP1* (CAGL0J02508g), *AWP2* (CAGL0K00110g), *AWP3* (CAGL0J11891g), *AWP4* (CAGL0J11990g), *AWP5* (CAGL0K13024g), *AWP6* (CAGL0G10175g), *AWP7* (CAGL0C00209g), *EPA1* (CAGL0E6644g), *EPA3* (CAGL0E06688g), *EPA6* (CAGL0C00110g), *EPA7* (CAGL0C05643g), *EPA22* (CAGL0K00170g) as well as for the housekeeping genes *URA3* and *ACT1* were taken from Kraneveld *et al.* [32].

For qRT-PCR, whole RNA was isolated using the RNeasy Mini Kit (Qiagen, Hilden, Germany) from cells that were harvested from overnight cultures (16 h) and broken using a Fast Prep 120 machine. RNA concentration was measured using a Nanodrop spectrophotometer (NanoDrop Technologies Inc., Wilmington, DE). Five hundred ng of total RNA was applied to DNase digestion using a TURBO DNAfree-kit without RNA Cleanup (Ambion, Austin, TX) to remove possible genomic DNA contamination. Fifty ng of DNA-free total RNA was reverse transcribed to complementary DNA (cDNA) using a first strand cDNA synthesis kit (Fermentas, St. Leon-Rot, Germany) with Oligo(dT)18 primers, to generate full-length transcripts. For each strain, RNA isolation and subsequent cDNA synthesis was performed on two independent cultures, which were analyzed in duplicate. Real-time qPCR was performed in 96-wells plates using a LC480-II light cycler (F. Hoffmann-La Roche, Basel, Switzerland). Real-time qPCR reactions were executed as described previously [32]. Adhesin genes subjected to this analysis are listed below table 2. Subsequently, a melting curve analysis was performed to test if any non-specific PCR products were generated. The qPCR reactions of the samples had similar efficiencies as the standard curve. Therefore, gene expression levels from the standard curve were used to extrapolate the gene expression levels of the samples and these were then normalized with the geometric mean of the results from the two housekeeping genes *ACT1* and *URA3*.

### Susceptibility Testing

Susceptibility testing towards cell wall- and membrane-perturbing agents (Congo red [CR] (Sigma-Aldrich, Saint Louis, MO), Blancophor P [BLP] (Prechel GmbH, Schwetzingen, Germany) and SDS (Sigma-Aldrich) was performed by broth microdilution. To prepare the inoculum, cells from agar plates were added to 0.9% NaCl to a density of 0.5 McFarland (5×10^6^ cells•ml-1), diluted 20-fold in YPD and 100 µl added to an equal volume of YPD containing CR or BLP. Plates with two-fold serial dilutions of CR or BLP were incubated at 30°C for 48 h and the cell densities were measured in a spectrophotometer. For SDS, about 1×10^4^ cells from overnight cultures in YPD were added to plates containing two-fold serial dilutions of SDS in YPD and grown for 17 h. Caspofungin E-tests were applied as outlined by the manufacturer (BioMerieux, Marcy l’Etoile, France). Calcofluor white susceptibility was tested in a spot assay, by placing 5 µl drops of ten-fold serial dilution onto YPD agar containing 250 µg•ml^−1^ Calcofluor white (Sigma-Aldrich). Average values were calculated from three independent experiments.

### Cell Lysis Assays

Cells from an overnight culture were diluted to an OD_600_ of 0.1 and incubated for further a 4 h. These log-phase cells were harvested, washed, resuspended in 50 mM Tris-HCl, pH 7.5 to an OD_600_ of 1, and Zymolyase 20T (AMS Biotechnology ltd., Frankfurt, Germany) was added to a final concentration of 4 units•ml^−1^. Cell lysis was monitored at room temperature by measuring the OD_600_ every 10 minutes for 120 min. Average values were calculated from three independent experiments.

### FACS Measurement

For CR staining of cell wall glucan, cells from 1 ml of an overnight culture were harvested and washed twice with PBS. Cells were resuspended in 1 ml of 1 M NaOH, boiled for 5 min, washed again twice with PBS and incubated for 30 min at room temperature in 300 µl CR staining solution (200 µg•ml^−1^ CR). After washing, stained cells were resuspended in 500 µl water.

For wheat-germ agglutinin (WGA) staining of cell wall chitin, cells from 1 ml of an overnight culture were harvested and washed twice with PBS. Cells were resuspended in 1 ml of 1 M HCl, boiled for 5 min and then washed again twice with PBS. Cells were resuspended in 300 µl WGA staining solution (50 µg•ml^−1^ WGA–Alexafluor 647 conjugate (Invitrogen, Eugene, OR)), incubated for 30 min at room temperature and washed again twice. Stained cells were resuspended in 500 µl water.

Flow cytometric analyses were performed with a FACSCalibur flow cytometer using CellQuestPro software (BD-Biosciences, Franklin Lakes, NJ). All values were calculated as the mean average fluorescence intensity of 12000 events in three independent experiments.

### Plastic Adhesion Assay

For a qualitative comparison of adhesion capacity, 25 µl of an overnight culture in YPD were spotted onto polystyrene Petri dishes (Greiner bio-one, Frickenhausen, Germany) and incubated at 30°C in a wet chamber. After 24 h, non-adherent cells were removed with sterile water and the spots were stained with 0.1% [w/v] Crystal violet (CV) for 5 min. Excess of CV solution was washed away and the plates were dried.

For quantitative determination, cells were grown in YPD for 24 h, after which the OD_600_ was adjusted to 2, and 50 µl of these cell suspensions were transferred into 150 µl fresh medium in polystyrene 96-wells plates (Greiner bio-one). After 24 h of incubation at 30°C, non-adherent cells were washed out with sterile water and the adherent cells were stained with 0.1% CV for 5 min. Excess CV was washed away and the cells were lysed with a solution containing 50% ethanol and 1% SDS. Measurements of adherent cells were achieved by measuring the OD_630_. All strains were tested three times with measurements being performed in triplicate.

### Silicone Adhesion Assay

To measure adherence to silicone surfaces, 1×1 cm silicone pieces (AMT Aromando, Düsseldorf, Germany) were incubated for 24 h at 37°C in 12-wells plates containing 2 ml YPD inoculated at a cell density of 0.08 McFarland with the *C. glabrata* strains. To remove unbound cells by aspiration, silicone pieces were transferred into fresh 12-wells plates containing 1 ml PBS. Bound cells were scraped off, resuspended in 1.2 ml PBS, and quantified by measuring the OD_600_. All values were calculated from three independent experiments.

### Statistical Analyses

Statistical significance for phenotypic tests was calculated using Student’s t-test. Differences in adhesin gene expression levels among CBS138 strains were statistically tested by ANOVA (SPSS 15.0). Separate Student’s t-tests were conducted to examine which genes were significantly reduced in individual strains. A *p*-value <0.05 in all cases was considered to be significant.

## Results

### Isolates of the CBS138 Reference Strains can be Divided into Three Distinct Karyotypes

**Figure 1:**
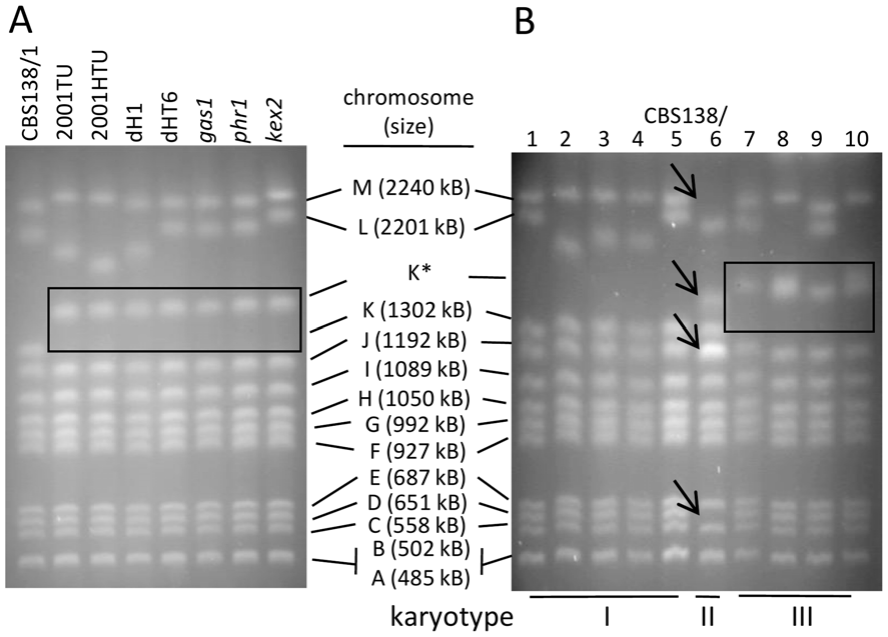
Electrophoretic karyotyping. **** PFGE analysis of CBS138-derived gene deletion progenies and ten different CBS138 isolates (A) CBS138-derived gene deletion progenies of *C. glabrata*, which are based on the trp^-^ ura^-^ and his^-^ trp^-^ ura^-^ auxotrophic strains that were produced by Kitada *et al.* [7]. All these strains carry the ChrK aberration (boxed, K*). (B) Variants of the parental CBS138 strain obtained from various laboratories. The isolates fall into three distinct karyotypic patterns, including the unaltered CBS138 reference karyotype (karyotype I), the karyotype observed in the Kitada-lineage (boxed, karyotype III), but also one additional, previously unobserved karyotype (arrows pointing to altered chromosomes, karyotype II).

Comparisons of karyotypes within the published lineages of genetically engineered progenies based on CBS138, including (i) the auxotrophic strains described by Kitada *et al.* [7], (ii) the auxotrophic strains described by Weig *et al.* [8] and (iii) three gene deletion strains [8], [33] show that the ChrK aberration, which was previously recognized in dH1 [22] is present in all of those strains (Figure 1A). To further back-track the aberration, we obtained isolates of the unmanipulated parental strain CBS138 from the stocks of nine scientific laboratories working scientifically with this strain (here termed CBS138/2–10) and the original isolate directly from the Centraalbureau voor Schimmelcultures (here termed CBS138/1). To exclude that isolates included in this study were accidently mixed up during propagation, MLST-analysis was performed. These data showed that the collected isolates are indistinguishable by MLST sequencing. Within the limits of the method, this confirmed that the analyzed strains are isogenic and of true common origin from the same culture collection isolate CBS138-1.

Based on their karyotypic profiles, the ten different isolates showed three distinct patterns, one of them being the original CBS138 karyotype (Figure 1B, karyotype I), a second the Kitada-lineage karyotype (Figure 1B, karyotype III) and a third karyotype in strain CBS138/6 (Figure 1B, karyotype II). The latter showed structural and numerical aberrations in several chromosomes (loss of the ChrD and ChrM bands, a novel band migrating above the ChrK band, and a signal intensification of the chromosome J band), indicating a possible recombination of ChrM with ChrD. In addition, variations in the two largest chromosomes ChrL and ChrM were observed in isolates among all three karyotypic groups.

### Karyotypes Correlate with Cell Wall Phenotypes

**Figure 2:**
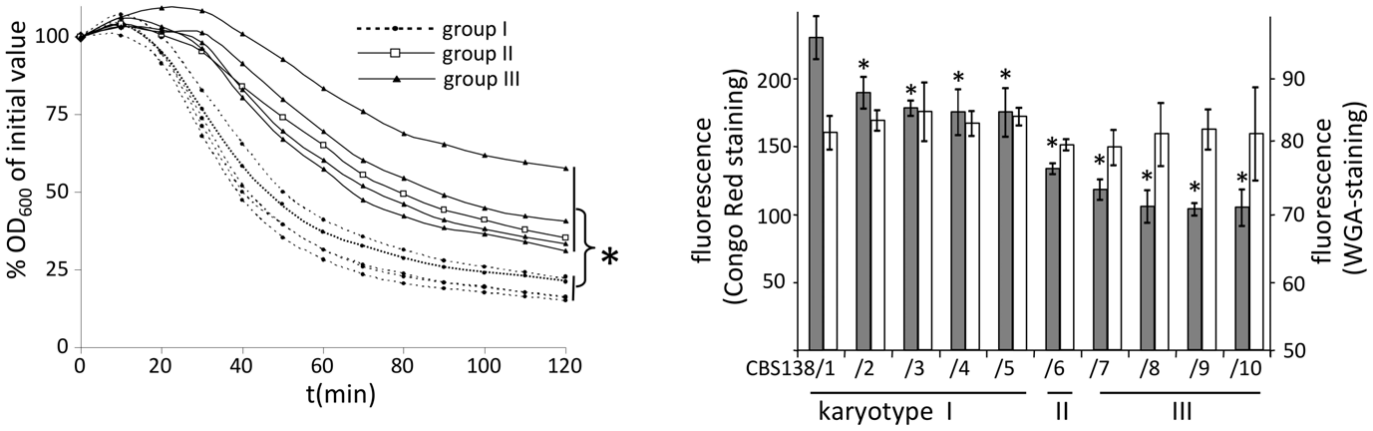
Karyotypes of different CBS138 derivates correlate with cell wall phenotypes. (A) Zymolyase-induced cell lysis is CBS138-karyotype dependent. Cell lysis rates by zymolyase activity are significantly (p = 0.009, indicated by asterisk) increased in karyotype I isolates as compared to karyotypes II and III. (B) Measurements of relative glucan and chitin levels showed that glucan content was CBS138-karyotype dependent. FACS measurements of CR-stained (dark bars) or WGA-stained (white bars) cell wall carbohydrates showed that karyotype I isolates had significantly higher content of alkali-insoluble glucan (CR-staining) than both karyotype II and III isolates (p_(I vs. II)_ = 0.0016; p_(I vs. III)_<0.0001). Within karyoptype I isolates, CBS138/1 showed significantly higher CR-staining compared to the other members of this group and significant differences of all individual strains in comparison to the reference strain CBS138/1 are indicated by asterisks (p<0.05). Content of acid-insoluble chitin (WGA-staining) was not significantly altered between the groups.

The analysis of cell wall-related phenotypes showed that the ten isolates can be grouped into three distinct phenotypic sets (Table 3, Figure 2), which mirror the karyotypic subgroups: the three karyotype groups have significant differences in tolerance towards the cell wall-perturbing agents Blancophor P (BLP), Calcofluor white and Congo red (CR), with karyotype I isolates being highly susceptible, CBS138/6 showing intermediate growth, and karyotype III isolates showing comparatively reduced susceptibility. Karyotype III isolates also have a reduced susceptibility towards caspofungin. Furthermore, upon treatment with the cell wall-degrading enzyme zymolyase, karyotype I isolates showed faster cell lysis than karyotypes II and III. Karyotype I isolates also showed the highest susceptibility to the membrane-perturbing agent SDS, however, in this assay CBS138/6 appeared more resistant than the karyotype III strains.

**Table 3:** Karyotypes of different CBS138 derivates correlate with their susceptibilities towards cell wall- or membrane perturbing agents.

Strain CBS 138/	Karyotypic group	Caspofungin	Calcofluor white	Congo Red	Blancophor P	SDS
		E-test MIC (mg/L)	Growth in spot assay	MicrodilutionMIC_80_ (mg/L)	MicrodilutionMIC_80_ (mg/L)	MicrodilutionMIC_80_ (mg/L)
1	I	0.094	none	4	0.125	0.031
2		0.094	none	4	0.25	0.016
3		0.094	none	4	0.125	0.016
4		0.094	none	4	0.25	0.016
5		0.094	none	4	0.125	0.016
6	II	0.094	intermediate	16	1	0.25
7	III	0.125	full	32	2	0.063
8		0.125	full	32	2	0.063
9		0.125	full	64	4	0.063
10		0.125	full	32	2	0.063

### The Groups Display Altered Cell Wall Compositions

The cell wall-related phenotypes suggested an altered cell wall composition between the groups. Therefore, the main cell wall components were examined using CR (detecting glucan) and WGA (detecting chitin) in flow-cytometric assays (Figure 2B). These analyses of the relative cell wall glucan and chitin contents confirmed that the glucan content of the individual strains was converse to the respective tolerance against cell wall perturbing agents. Karyotype I isolates exhibited an elevated glucan-content, which was highest in the original CBS138/1 strain and was at similar levels in strains CBS138/2–5. Again, CBS138/6 could be categorized as intermediate, whereas karyotype III isolates CBS138/7–10 had the lowest glucan content. In contrast, there were no detectable significant differences in levels of acid-insoluble cell wall chitin.

### Karyotypic Groups Correlate with Different Cell Surface Properties

**Figure 3:**
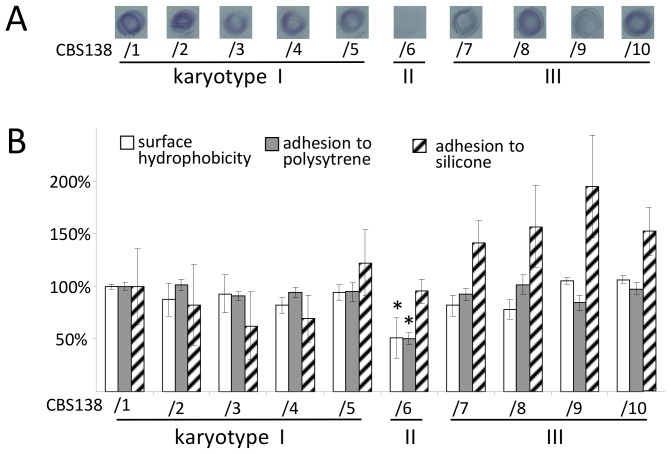
Cell surface characteristics. (A) Qualitative determination of adherence to polystyrene. (B) Quantitative determination of hydrophobicity (white bars), adherence to polystyrene (grey bars) and adherence to silicone (striped bars). All values are normalized against the values of isolate CBS138/1. Statistically significant differences were observed for karyotype II measurements of hydrophobicity and polystyrene adherence as compared to karyotype I and III (p_(II vs. I)_ = 0.0015; p_(II vs. III)_ = 0.0030 and p_(II vs. I)_<0.0001; p_(II vs. III)_ = 0.0008, respectively), and for karyotype III for silicone adhesion (p_(III vs. I)_ = 0.0001; p_(III vs. II)_<0.0001). The significant differences of CBS 138/6 (karyotpe II) in comparison to the reference strain CBS138/1(karyotype I) are indicated by asterisks (p<0.05).

**Figure 4:**
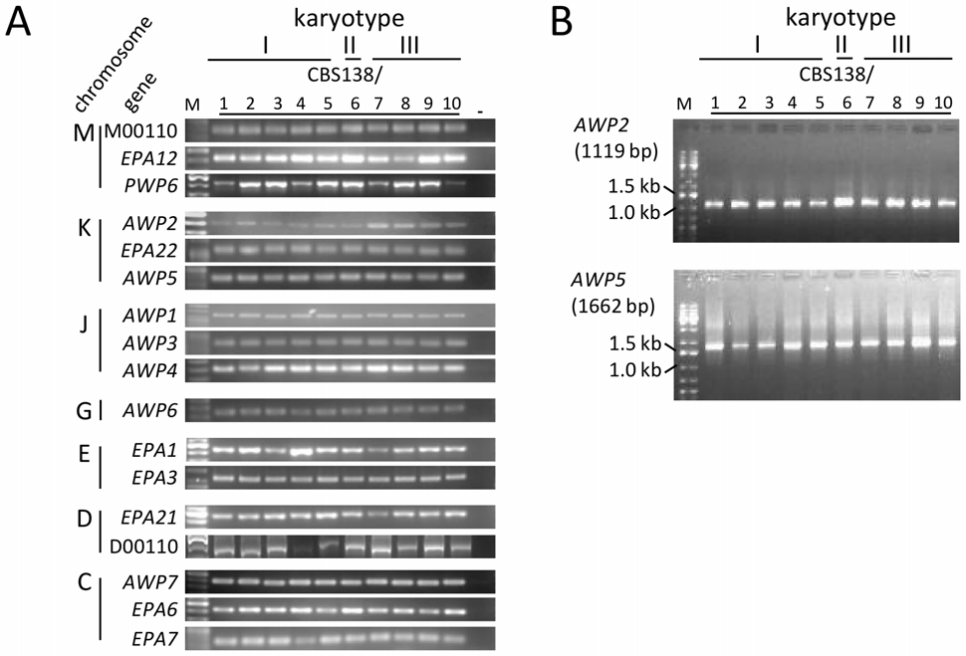
Genomic analysis of gene presence and repeat length of selected adhesin genes in CBS138 derivates does not reveal alterations. (A) The presence of selected adhesin genes in the genomes of the different isolates was analyzed by PCR. All genes tested were present in all strains. (B) PCR analysis of the repeats-containing C-terminal regions of *AWP2* and *AWP5*. No differences were detected.

Qualitative as well as quantitative analyses of cell wall adhesion properties demonstrated that, in addition to the cell wall-related phenotypes described above, the karyotype II isolate (CBS138/6) exhibited a significantly reduced adhesion capacity to polystyrene surfaces, and its surface hydrophobicity was also significantly reduced. Furthermore, karyotype III isolates exhibited significantly increased adherence to silicone (Figure 3). As the karyotype of CBS138/6 suggested a recombination event of ChrM and ChrD, and the karyotypes of CBS138/7–10 suggested a recombination involving ChrK, we investigated whether the subtelomeric genes of previously predicted adhesins were still present on these altered chromosomes [24]. Using genomic DNA, we also tested if adhesin genes on other chromosomes (C and J), whose products have previously been found in the cell wall of *C. glabrata* [24], [32], were still present in the genomes. To confirm the specificity of the PCRs conducted, all DNA products obtained from isolate CBS138/6 were sequenced (data not shown). For all adhesin genes tested, specific products could be obtained in all strains, confirming that the observed modifications of adhesion capacity was not based on gene loss during chromosomal recombination events (Figure 4A). Previously, a correlation between the number of repetitions of intragenic tandem repeats (megasatellites), which often occur in the C-terminal parts of adhesin genes, and adhesion properties has been shown [34], [35]. However, additional PCR analysis indicated that there were no alterations in the number of VSHITT signature-containing repeats in *AWP2* and *AWP5* (Figure 4B) of the CBS138 derivates that might explain the differences that were found in the adhesion assays.

**Figure 5:**
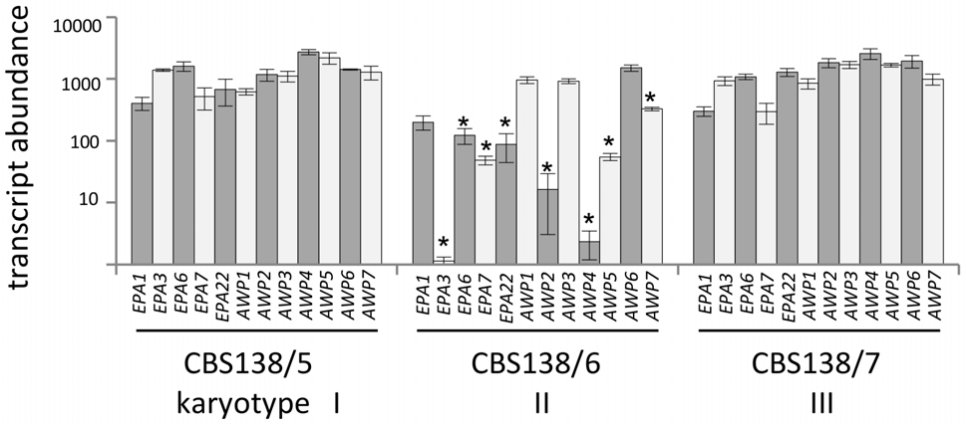
CBS138 derivates show differences in adhesin gene expression. Gene expression analysis of adhesin genes in selected strains, representing the three karyotypes (CBS138/5 (karyotype I), 6 (karyotype II), and 7 (karyotype III)), revealed that adhesins are differentially expressed between the strains (ANOVA). T-tests showed significant reduced adhesin gene expression for CBS138/6 compared to CBS138/5 and CBS138/7 for *EPA3*, *EPA6*, *EPA7, EPA22, AWP2*, *AWP4*, *AWP5* and *AWP7* (indicated by asterisks (p<0.05)).

Finally, gene expression analysis of adhesin genes in selected strains representing the three karyotypes (CBS138/5 (karyotype I), 6 (karyotype II), and 7 (karyotype III)), revealed that adhesins are differentially expressed. Noteworthy, strain CBS138/6 shows a significantly reduced expression of numerous adhesin genes in the stationary growth phase: e.g. *EPA3*, *EPA6*, *EPA7*, *EPA22*, *AWP2*, *AWP4, AWP5* and *AWP7* (Figure 5). These differences are not based on unequal fitness of the derivates as confirmed by growth curve analyses (data not shown).

## Discussion

The *C. glabrata* CBS138/ATCC2001 type strain, originally isolated from human feces, was selected for the genome sequencing project of this species by the Génolevures consortium [6], [36]. Genetically engineered auxotrophic progenies of this strain are now widely used by the scientific community as molecular tools to analyze the function of genes of interest in this fungus (e.g. [6], [7], [8], [33], [37], [38], [39]). Recently, Muller *et al.* [22] observed that the CBS138-based histidine auxotrophic laboratory strain dH1 [8] harbors a chromosomal rearrangement involving ChrK (chromosome 11). Our karyotypic comparison of dH1-derived strains (including the auxotrophic parental strains produced by Kitada *et al.* [7], as well as published mutants derived from the dH1 and dHT6 strains [8]) showed that this aberration is already present in the parental strains (Figure 1), and that the karyotypic alterations were stable through the gene deletion processes of the *GAS1*, *GAS2* [8], and *KEX2* [33] genes. To further back-track the aberration and to analyze genetic plasticity as well as the phenotypic diversity that might have been induced under laboratory conditions, we compared nine samples of the unmanipulated parental wild type strain CBS138 (CBS138/2–10) provided by different research laboratories to their common origin (CBS138/1) obtained directly from the CBS. The different derivates of CBS138 were indistinguishable by multi locus sequence typing, but are separated into three distinct karyotypic patterns. Besides frequent length polymorphisms of chromosomes L and M, which harbor the highly variable rDNA loci and are reportedly prone to rearrangements and length polymorphisms [16], [22], two karyotypic aberration patterns were found as compared to the karyotype of CBS138/1 (Figure 1): one of them matched with the observed pattern in the Kitada lineage (four samples; karyotype III), and the other represents a karyotype that had not been observed in CBS138 so far (sample CBS138/6, karyotype II). These additional chromosomal rearrangements in CBS138 most notably involved the chromosomes D, M (karyotype II) or K (karyotype III).

To analyze if these karyotypic alterations were associated with changes in cell wall characteristics, we submitted the ten different lineages to several common cell wall phenotypic tests including determination of susceptibility towards cell wall perturbing (Congo red, Calcofluor white, Blancophor P, Caspofungin) and membrane perturbing (SDS) agents, the lytic enzyme zymolyase, and we also analyzed the relative content of glucan and chitin in the cell wall. In these phenotypic studies, strains within a specific karyotypic pattern group were similar to each other; however, we observed significant phenotypic variations between the three groups. Karyotype pattern I isolates were highly susceptible towards cell wall and membrane perturbing agents (Table 3) and to cell lysis by zymolyase (Figure 2A) as compared to the other groups. Karyotype pattern III isolates showed slightly elevated MICs for Caspofungin. The isolate CBS138/6 had an intermediate phenotype in these assays except for the SDS sensitivity, which was lowest in this strain. The observed phenotypic alterations also correlated with changes in the relative amounts of alkali-insoluble cell wall glucan, suggesting that quantitative changes in cell wall carbohydrate composition contribute to the modified resistance to cell wall perturbing agents (Figure 2B).

With no selective pressure being applied in our studies, other remarkable observations were the significant loss of adherence to polystyrene and the concomitant loss of hydrophobicity in the derivate CBS138/6 (Figure 3). Previous studies have identified more than 65 genes of putative adhesins in the genome of *C. glabrata* CBS138 and the incorporation of the adhesin(-like protein)s Awp2–6, Epa3, and Epa6 was demonstrated in the cell wall of CBS138 using proteomic approaches [24], [32], [40]. Differential incorporation of adhesins and surface hydrophobicity was found to depend on the genetic background of the *C. glabrata* strains, the growth phase [24], and biofilm formation [32]. To study adhesin-expression in the CBS138 derivates in more detail, we first performed comparative qualitative and quantitative adhesion tests (Figure 3), and then applied real-time PCR assays to quantify the expression of all adhesins that were previously identified as part of the cell wall proteome of *C. glabrata* [24], [32]. These assays confirmed that the loss of adhesion to polystyrene and reduction of surface hydrophobicity in CBS138/6 is associated with a significantly reduced expression of many adhesins, most notably in *EPA3, EPA6, EPA7, EPA22, AWP2, AWP4 and AWP5* (Figure 5). However, the diminished expression of these genes in CBS138/6 is not simply based on the loss of the respective genes during the observed chromosomal rearrangements, as PCR analyses of genomic DNA confirmed the presence of these adhesin genes in all ten CBS138 derivates (Figure 4A). In a previous study, consistent with these new findings for CBS138/6, Epa6, Awp2 and Awp4 were found absent from the cell wall proteome of *C. glabrata* strain ATCC90876, which is also non-adherent to plastic and less hydrophobic as compared to CBS138/4 [24]. However, the binding substrates of the non-Epa adhesins to date is completely unknown and remains speculative.

All adhesin genes with a significantly altered expression in derivate CBS138/6 in this study are located at subtelomeric loci [40]. Therefore, the observed transcriptional regulatory events may involve epigenetic mechanisms such as *SIR*-dependent gene silencing [13], [41], [42]. In addition, many adhesins in *C. glabrata* are “megasatellite”-containing genes carrying long tandem repeats [12], [24]. These intragenic repeats contribute to functional variability and quantitative alterations in phenotypes such as adhesion, flocculation or biofilm formation in yeasts [34]. In the *C. glabrata* CBS138 genome sequence signatures (VSHITT) of Awp2 megasatellites are present in the adhesins Awp2 (4–5 repeats), Awp4 (15 repeats) and Awp5 (6 repeats) [24]. During DNA-replication addition or removal of repeat units can occur, leading to expansion or contraction of C-terminal low complexity stalk regions and influencing the adherence capacity of these proteins [35]. Consistent with this, we recently identified gene length variations in the repeat-containing regions of *AWP2* in clinical isolates (unpublished data). Analysis of the repeat regions in *AWP2* and *AWP5* did not reveal expansions or reductions of these regions in any of the ten CBS138 isolates, indicating that the altered surface properties of the derivates cannot directly be attributed to changes in the number of repeats in these two genes (Figure 4B). We did not succeed to amplify the repeat region of *AWP4*, possibly because of its much larger size. Also, analysis of the DNA translation of the genomic region spanning this gene suggests that it contains multiple frameshift errors due to sequencing mistakes.

In this study, we compared the karyotypes and phenotypes of different derivates of the laboratory strain *C. glabrata* CBS138. We show that significant plasticity is present not only *in vivo* but also *in vitro* under laboratory conditions without extreme selective pressure. The data point to mechanisms of evolution that affect both, genome-rearrangement driven variations and modifications in phenotypic properties of research interest, such as cell wall characteristics, susceptibility to antimycotic substances, surface hydrophobicity and adhesion. Although we have not yet observed any chromosomal aberrations which have occurred during *in vitro* mutagenesis in *C. glabrata* in our lab, these new data on the CBS138 reference strain stress the importance to carefully check genetically engineered mutants and parental strains for altered karyotypes and phenotypes. This plasticity should be seriously considered when strains are shared between different laboratories or when phenotypic data, e.g. of independently cloned mutants, are analyzed and compared. It also raises the question how strains should be maintained best and if standardized quality control protocols could be useful for the scientific community.
