# Correction: Gross Karyotypic and Phenotypic Alterations among Different Progenies of the *Candida glabrata* CBS138/ATCC2001 Reference Strain

**DOI:** 10.1371/annotation/b929fe83-8e7f-4e8a-986e-9a3e26d86c54

**Published:** 2013-06-07

**Authors:** Oliver Bader, Alexander Schwarz, Eefje A. Kraneveld, Marut Tangwattanachuleeporn, Pia Schmidt, Mette D. Jacobsen, Uwe Gross, Piet W. J. De Groot, Michael Weig

The fourth author's name was spelled incorrectly. The correct name is: Marut Tangwattanachuleeporn.

The correct citation is: Bader O, Schwarz A, Kraneveld EA, Tangwattanachuleeporn M, Schmidt P, et al. (2012) Gross Karyotypic and Phenotypic Alterations among Different Progenies of the Candida glabrata CBS138/ATCC2001 Reference Strain. PLoS ONE 7(12): e52218. doi:10.1371/journal.pone.0052218 

